# Transplantation Tolerance through Hematopoietic Chimerism: Progress and Challenges for Clinical Translation

**DOI:** 10.3389/fimmu.2017.01762

**Published:** 2017-12-22

**Authors:** Benedikt Mahr, Nicolas Granofszky, Moritz Muckenhuber, Thomas Wekerle

**Affiliations:** ^1^Department of Surgery, Section of Transplantation Immunology, Medical University of Vienna, Vienna, Austria

**Keywords:** tolerance, chimerism, allograft rejection, transplantation immunology, immunosuppression

## Abstract

The perception that transplantation of hematopoietic stem cells can confer tolerance to any tissue or organ from the same donor is widely accepted but it has not yet become a treatment option in clinical routine. The reasons for this are multifaceted but can generally be classified into safety and efficacy concerns that also became evident from the results of the first clinical pilot trials. In comparison to standard immunosuppressive therapies, the infection risk associated with the cytotoxic pre-conditioning necessary to allow allogeneic bone marrow engraftment and the risk of developing graft-vs.-host disease (GVHD) constitute the most prohibitive hurdles. However, several approaches have recently been developed at the experimental level to reduce or even overcome the necessity for cytoreductive conditioning, such as costimulation blockade, pro-apoptotic drugs, or Treg therapy. But even in the absence of any hazardous pretreatment, the recipients are exposed to the risk of developing GVHD as long as non-tolerant donor T cells are present. Total lymphoid irradiation and enriching the stem cell graft with facilitating cells emerged as potential strategies to reduce this peril. On the other hand, the long-lasting survival of kidney allografts, seen with transient chimerism in some clinical series, questions the need for durable chimerism for robust tolerance. From a safety point of view, loss of chimerism would indeed be favorable as it eliminates the risk of GVHD, but also complicates the assessment of tolerance. Therefore, other biomarkers are warranted to monitor tolerance and to identify those patients who can safely be weaned off immunosuppression. In addition to these safety concerns, the limited efficacy of the current pilot trials with approximately 40–60% patients becoming tolerant remains an important issue that needs to be resolved. Overall, the road ahead to clinical routine may still be rocky but the first successful long-term patients and progress in pre-clinical research provide encouraging evidence that deliberately inducing tolerance through hematopoietic chimerism might eventually make it from dream to reality.

## The Long-Lasting Journey of Tolerance

Transplantation is the treatment of choice for patients with end-stage organ failure (1, 2) as it improves their survival and quality of life (3). Nevertheless, long waiting lists, side effects of immunosuppressive medication, and limited graft survival still perpetuate the dream of tolerance. Initial enthusiasm was fueled in the 1950s by the observation of Medawar and Billingham that dizygotic twins of free martin cattle readily accept skin grafts from each other (4). They attributed this phenomenon to the coexistence of red blood cells from their siblings (mixed chimerism) as a result of a common placental circuit (5). Since fraternal erythrocytes also persisted in adult animals, they concluded that stem cells had to be exchanged which constantly give rise to short-lived red blood cells. Based on this observation, they demonstrated for the first time that tolerance to alloantigens can actively be acquired in neonatal mice by the intrauterine injection of allogeneic cells (6). Since then, a myriad of mouse studies established bone marrow transplantation as promising approach to achieve donor-specific transplantation tolerance (7). In the clinical setting, the usability of this approach was corroborated by anecdotal cases in which patients developed immunological tolerance to a renal allograft after having previously received a hematopoietic stem cell transplant from the same donor for a hematological disorder (8). Despite this knowledge, the establishment of tolerance inducing protocols as a clinical routine is still long in coming.

Three groups in the US from Harvard University, Stanford University and the association between the Universities of Louisville and Northwestern have pioneered the first steps toward clinical implementation. All three groups have elaborated distinct approaches with individual assets and drawbacks and so far, nearly 70 patients have been enrolled (9–11). Here, we discuss the current state of affairs of chimerism-based tolerance, what we have learned so far and which new challenges we have to face.

## Tolerance—Is it Worth It?

Continuous suppression of the immune system puts transplant recipients at risk of increased morbity and mortality through cardiovascular disease, *de novo* diabetes, dyslipidemia, and malignancies. By now, death with a functioning graft has become a leading cause of graft loss (2). Some of these side effects result from impaired immune surveillance while others constitute drug-specific toxicities of the immunosuppressive medication. Cyclosporine was initially celebrated as “wonder drug,” until it was realized that it is fairly toxic at higher doses (12). In the light of this, a substantial number of non-renal organ transplant recipients develop renal failure due to calcineurin inhibitor-toxicity (13). Apart from that, immunosuppressive drugs are ineffective in preventing late graft loss from chronic rejection (14) which is why long-term graft survival has improved only marginally over the last decades (2).

Since tolerance is expected to provide remedy, the search for the “Holy Grail” of transplantation has never ceased. To assess whether tolerance actually meets these high expectations, tolerant kidney transplant recipients have recently been compared to a matched cohort receiving conventional immunosuppression. The tolerant group experienced significantly longer initial hospital stays and more frequent readmissions leading to three times higher costs during the first year in comparison to conventional transplant recipients. In turn, tolerant patients required significantly less treatment for hypertension and none of them developed new-onset diabetes, dyslipidemia, or malignancy. In this survey, the continuous costs for medications of conventional patients exceeded those of tolerant patients after 10 years (15, 16). The sample size was small but this preliminary study emphasized the potential benefit of tolerance inducing protocols. Another group estimated the expected lifetime savings through tolerance induction for a 40-year old patient receiving a kidney from a human leukocyte antigen (HLA)-matched living donor to approximately 92.000$ (17). Besides, it should be taken into account that innovative treatment options become less expensive if employed as clinical routine, especially if they are also applicable to other medical fields. In this regard, mixed chimerism becomes increasingly attractive as treatment option for autoimmune disorders (18, 19).

In addition, tolerant patients evidently enjoy a higher quality of life (16), which is not only a matter of convenience but also correlates with reduced morbidity and mortality (20). The discomfort resulting from the immunosuppressive therapy increases the probability of non-adherence which in further consequence leads to decreased donor organ survival. Considering that kidney transplant recipients take a median of 15 capsules a day (21), it appears not surprising that non-adherence is estimated to occur roughly in a third of all transplant recipients (2).

Tolerance inducing protocols are currently measured against kidney transplant recipients receiving conventional immunosuppressive therapy. One-year graft survival rates of over 90% and half-lives of 16 years set the bar fairly high (22, 23). Innovative strategies aiming to improve patient and organ survival will further increase the high demands for tolerance inducing strategies. New algorithms have been developed to allocate best-quality organs to those recipients expected with the highest survival (24) and paired living kidney donation programs optimize allograft survival (25). Furthermore, the long-lasting supremacy of calcineurin inhibitors has recently been challenged by the advent of costimulation blockers in the clinical setting. Belatacept was associated with enhanced overall patient and graft survival in subsets of patients, improved kidney allograft function and avoided major side effects of calcineurin inhibitors (26).

In summary, tolerance is still of great value, particularly in the longer run, but the question remains whether it can be achieved at an acceptable prize in terms of safety. Tolerance would certainly obviate the common risks of immunosuppressive medication but at the same time expose patients to other serious hazards.

## Graft-vs.-Host Disease (GvHD)—The Problem Child of Chimerism

When it comes to clinical translation, patient safety takes the highest priority. Therefore, the conditioning necessary to achieve allogeneic bone marrow engraftment is a matter of great concern. In contrast to conventional kidney allograft recipients, patients receiving hematopoietic stem cell transplantation for the purpose of tolerance induction are additionally exposed to a considerable risk of developing GvHD. Approximately 15% of patients undergoing hematopoietic stem cell transplantation for hematological diseases succumb to GvHD (27). The incidence and severity of acute GvHD can directly be correlated with the degree of HLA mismatch (28). Since tolerance is particularly desirable for HLA-mismatched recipients, the prevention and treatment of GvHD is a delicate issue. The occurrence of GvHD is highly dependent on the recipient conditioning and the composition of the allograft. Therefore, we will discuss which efforts the individual groups have made to minimize the risk of GvHD in the clinical pilot trials of chimerism-based tolerance.

### Irradiation

Ionizing irradiation mostly affects mitotically active cells by causing breaks in DNA double strands. The cells of the hematopoietic system and the gastrointestinal tract exhibit a high degree of proliferation and are, thus, particularly sensitive to irradiation (29). Accordingly, high doses of total body irradiation (TBI) do not only obliterate the bone marrow compartment as required but also cause damage to the gastrointestinal tract. Bacterial molecules leaking from the injured gut elicit the release of inflammatory cytokines (e.g., TNF-α, IL-1, IL-6) through activation of innate immune receptors which promotes the induction of acute GvHD (30). In current clinical practice, the total dose is typically partitioned into lower doses to allow normal tissues to partially recover between the individual fractions (31). In addition, current effort is focused on the targeted neutralization of major inflammatory cytokines (32).

The clinical trials from the Stanford group are built upon a specific form of irradiation which was actually designed for the treatment of lymphomas (33). At this, irradiation is restricted to lymphatic tissues, including supradiaphragmatic lymph nodes, thymus, subdiaphragmatic lymph nodes, and spleen. Since a large part of the marrow volume is outside the radiation fields, recovery of blood elements occurs without severe neutropenia or thrombocytopenia (34). Total lymphoid irradiation (TLI) depletes the majority of lymphocytes within the targeted tissue end enriches the residual cells for CD8^+^ dendritic cells and natural killer T cells (NKT) cells. CD8^+^ dendritic cells prompt NKT cells to secrete IL-4 which prevents lethal GvHD through the expansion of donor Tregs (35, 36).

The group from Louisville adapted a conditioning regimen that was originally elaborated at the Johns Hopkins University for hematological disorders. This approach uses a single dose of 200 cGy TBI together with high-dose cyclophosphamide post-transplant to purge proliferating alloreactive T cells while sparing Tregs (37, 38). In this way, the increased proportion of donor Tregs prevents GvHD (39). Notably, the groups from Stanford and from Louisville both aim at increasing the number of donor Tregs to prevent GvHD. Donor Tregs have a vital role in reducing GvHD (40) and the infusion of donor Tregs has likewise been reported to prevent GvHD in the clinic (41).

The group from Boston attempted to reduce irradiation-related toxicities by specifically targeting those mechanisms resisting allogeneic bone marrow engraftment. In the murine setting, it was observed that depleting T cells with monoclonal antibodies allowed reducing myeloablative TBI. However, chimerism remained transient unless high doses of TBI were used (42), since these monoclonal antibodies efficiently depleted T cells in the periphery but did not reach T cells in the thymus. Hence, they combined T cell depleting antibodies with the targeted irradiation of the thymus to further decrease the required TBI to a non-myeloablative dose (43). Despite being successful in the murine setting, this regimen failed to induce stable mixed chimerism in non-human primates but instead led to transient chimerism. Nevertheless, the conditioning was sufficient to achieve tolerance to renal allografts across major histocompatibility complex (MHC) barriers as long as the kidney was transplanted before the loss of peripheral chimerism (44).

To exploit hematopoietic stem cell transplantation as clinical routine for tolerance induction it would be desirable, or indeed necessary, to avoid any form of irradiation. Several approaches have been elaborated in the murine setting to eliminate the cytotoxic preconditioning necessary to achieve allogeneic bone marrow engraftment. Costimulation blockade in the form of α-CD40L and CTLA4-Ig can only obviate the need for cytoreductive conditioning when clinically unrealistic marrow doses are administered (45, 46). The required bone marrow dose can be reduced through the addition of rapamycin or α-LFA-1 to the conditioning (47, 48). By contrast, conventional, clinically obtainable, doses of fully allogeneic bone marrow engraft in non-irradiated mice under costimulation blockade and rapamycin if *in vitro* activated Tregs from the recipient are administered at the time of donor bone marrow transplantation (49). An adjusted version of this approach has recently confirmed the feasibility of Treg therapy to enhance bone marrow engraftment in non-human primates (50). As clinical trials deploying Treg therapy without concomitant bone marrow transplantation are already underway, clinical translation of the combined cell therapy appears possible in the near future (51). Alternatively, the pro-apoptotic molecule ABT-737 synergized with α-CD40L and cyclosporine to induce chimerism and tolerance without the need for any cytoreductive conditioning (52). Both approaches, however, rely on CD40L blockade which is currently not available in the clinic due to unacceptable prothrombotic toxicities of conventional α-CD40L mAbs (53). In order to circumvent the unacceptable side effects of the original conventional α-CD40LmAbs, efforts were made to target its binding partner CD40 which is not expressed on thrombocytes. The humanized α-CD40 antibody ASK1240 has already been tested in a phase 2 trial of *de novo* kidney transplant recipients. The preliminary results, however, suggest a disappointing efficacy of CD40 blockade in a calcineurin inhibitor free regimen (54). Recently, next generation α-CD40L antibodies lacking thromboembolic side effects have shown promise in preclinical development and might become an option for use in tolerance protocols in the future (55).

### Graft Composition

The identification of donor T cells as the driving force of GvHD conveyed the idea that transplanting purified stem cells could promote engraftment while avoiding GvHD. But it was soon realized that highly purified mouse stem cells would only engraft in MHC-matched but not -mismatched recipients (56). This failure of purified stem cells to engraft was traditionally ascribed to their rejection by host immune cells. However, it could also be envisioned that non-stem cell components contained within the donor bone marrow compartment are required to facilitate stem cell engraftment in allogeneic recipients. Therefore, the group from Louisville set themselves the task to prove this latter assumption and to identify such a cell population that facilitates bone marrow engraftment without causing GvHD. Adding selected donor cell populations to a mixture of T cell depleted syngeneic and allogenic bone marrow revealed a heterogeneous mixture of cells expressing CD8^+^ albeit without a T cell receptor to promote stem cell engraftment (57). Further characterization of these murine “facilitating cells” unmasked plasmacytoid-precursor dendritic cells, B cells, granulocytes, as well as monocytes (58). Recipients of hematopoietic stem cells and “facilitating cells” displayed an increased RNA expression of GITR, CTLA4, and Foxp3 in the spleen 28 days post transplantation (59). In a subsequent study, the authors observed that CD8^+^ plasmacytoid precursor DCs were primarily responsible for the induction of antigen-specific Foxp3 Tregs. These induced Tregs were able to enhance stem cell engraftment and to suppress alloreactive T cells *in vitro* (60).

Human “facilitating cells” are composed of two equally divided cell populations which can be differentiated on the basis of their CD56 expression. Most CD56^bright^ cells are CD11c^+^ CD11b^+^ and exhibit a dendritic morphology. The majority of CD56^neg^ cells expresses CD3ε and displays a lymphoid shape. Both cell populations express the chemokine receptor CXCR4 which promotes homing to the bone marrow compartment (61). Although it remains speculative, it is reasonable to assume that CD8^+^ TCR^−^ CD56^bright^ “facilitator cells” and CD8^+^ DCs enriched through TLI share common features. The identification of this cell population constitutes the basic building block for the clinical trials of the Louisville/Northwestern group. A cell product containing hematopoietic stem cells and “facilitating cells” (also designated FCRx) is engineered through a proprietary, undisclosed procedure from G-CSF mobilized donor peripheral blood stem cells. The cell product is usually cryopreserved until it is infused 1 day after renal transplantation (62).

As already mentioned, the group from Stanford employed a regimen that was originally developed for patients with hematological malignancies. The key change for patients without malignancies was the alteration of graft composition in order to achieve mixed instead of full chimerism. While patients with malignancies received unmanipulated mobilized blood stem cells containing a high number of T cells (2–3 × 10^8^/kg), kidney recipients are transplanted with column enriched CD34^+^ cells supplemented with low numbers of T cells (1 × 10^6^/kg) (36). Under these circumstances kidney recipients have to be HLA matched in order to achieve stable mixed chimerism. Accordingly, this approach is only applicable to a restricted cohort of patients which enjoys anyhow best survival rates with current standard of care immunosuppressive treatment. However, the group from Stanford is currently conducting a clinical trial in the effort to determine the optimal graft composition for haploidentical donors (11).

The group from Boston transplants unseparated iliac crest marrow on the day of kidney transplantation, based on their experience from non-human primate studies (63). The acquisition of mobilized blood stem cells provides more comfort for the donors but mobilized blood stem cells have distinct biological characteristics that might affect their ability to induce tolerance. In a murine model, peripheral blood stem cells had a lower capacity to induce mixed chimerism and tolerance than conventional bone marrow due to the higher number of donor T cells (64), which can trigger rejection in an IL-6-dependent manner (65). From a clinical point of view, peripheral blood stem cell transplants are associated with a higher risk of acute and chronic GvHD but reduce the risk of graft failure owing to higher engraftment rates (66, 67). On the other hand, G-SCF mobilized stem cells upregulate CD47 to evade macrophage killing providing a possible explanation for their superior engraftment rates (68). Transplanting bone marrow and kidney at the same time requires the recipient conditioning to begin 6 days earlier which restricts this application to living donor transplant recipients. To extend their protocol to deceased donors the Boston group is currently endeavored to develop a “delayed tolerance” protocol. In this case, the recipients would first undergo kidney transplantation with conventional immunosuppression and subsequently receive cryopreserved bone marrow from the same donor. In non-human primates, kidney transplantation prior to bone marrow transplantation enhances the pool of alloreactive memory T cell responses, thus necessitating substantial CD8 T depletion to achieve mixed chimerism and tolerance (69, 70).

### Chimerism Type

The type of chimerism (full vs. mixed, durable vs. transient) is a determining factor for the risk of GvHD. Murine studies indicated that there is a considerably greater chance of developing GvHD in stable full chimeras than in stable mixed chimeras (71). Apart from that, animal studies predict that stable mixed chimerism would also offer other advantages over full chimerism. The induction of mixed chimerism requires less toxic pre-conditioning and full chimeras display impaired immune responses resulting from the discrepancy between positive selection of T cells by host thymic epithelial cells and antigen presentation by peripheral donor antigen-presenting cells (72). However, the establishment of stable mixed chimerism in the clinic remains a formidable challenge. Therefore, it seems noteworthy to discuss the different forms of chimerism achieved by the individual groups.

The Northwestern group aims to achieve full chimerism which provides a stable state of tolerance although at the expense of a risk for GvHD. If stable chimerism is achieved, the patient is likely to maintain robust tolerance. On the downside, two cases of GvHD have been reported, one of which was fatal. Moreover, all patients had severe neutropenia (absolute neutrophil count <500 cells/mm^3^) and 11 developed severe bacterial or fungal infections (10, 73).

The group from Stanford University aims to achieve stable mixed chimerism which is generally associated with a reduced risk of GvHD. However, thus far persistent mixed chimerism has solely been achieved in HLA-matched recipients who compromise only a minor patient cohort. Mixed chimerism is still difficult to achieve when HLA barriers are crossed. Anyhow, their conditioning showed a low incidence of adverse events as compared with other tolerance inducing strategies using cyclophosphamide and/or TBI (11).

The investigators from Boston consider GvHD as an inacceptable complication (44) for non-malignant patients and, therefore, intend to achieve tolerance through transient chimerism. Transient chimerism basically eliminates the risk of developing GvHD but exhibits a reduced stability of allograft tolerance. In non-human primate studies, 20–30% of the transiently chimeric recipients eventually developed antibody-mediated rejection during long-term follow-up (74). Similar complications occurred in the clinical trials wherefore the group had to adjust the protocol. Furthermore, all patients developed transient pancytopenia (75) and nine patients developed an engraftment syndrome with acute renal endothelial injury manifested by a creatinine rise during marrow recovery (76).

## Current State of Affairs

### Boston Group

In their first trial, the group from Boston enrolled patients with end-stage renal disease resulting from multiple myeloma. These patients received a combined kidney and marrow transplantation from a HLA identical sibling donor. Ten recipients have been reported with follow-up times of up to 17 years. The conditioning consisted of cyclophosphamide (60 mg/kg; days −6 and −5), α-thymocyte globulin (ATG) (15–20 mg/kg; days −1, 1, 3, and 5) and thymic irradiation (700 cGy; day −1). Maintenance therapy in the form of cyclosporine was administered and discontinued as early as 73 days post-transplant in the absence of GvHD. Five patients have been completely off immunosuppression for 5–17 years providing the first proof of concept that tolerance in humans can deliberately be achieved through bone marrow transplantation. Recently, the inclusion criteria have been extended to other hematological disorders and cyclophosphamide has been replaced by 400 cGy TBI after severe cardiac toxicity occurred in one patient (77, 78).

The group from Boston modified the protocol in order to make it accessible also for patients with haploidentical donors. Cyclophosphamide was administered before transplantation at lower doses (14.5 mg/kg; days −6 and −5) and at higher doses (50 mg/kg; days 4 and 5) after transplantation. Thymic irradiation was substituted by 200 cGy TBI and cyclosporine by tacrolimus and mycophenolate mofetil. After the first patient experienced graft rejection, ATG was replaced by fludarabine (24 mg/m^2^; days −6 to −2). The second patient tolerated the protocol fine, while the third patient deceased due to fludarabine-related neurotoxicity. Therefore, the dose of fludarabine was reduced from 5 to 3 days (24 mg/m^2^/days −4 to −2) and the duration of individual dialysis sessions was extended. So far the transplant course of the forth patient was uncomplicated and he is off drugs 6 months post transplantation (77–79).

With the experience of the HLA-matched myeloma patients, the Boston group pursued their approach in patients without concomitant malignancies. The patients received a kidney and iliac crest marrow transplantation from haploidentical donors after thymic irradiation (700 cGy, day −1) and a short course of cyclophosphamide (60mg/kg/days −5 and −4). In contrast to myeloma patients, ATG was replaced by the monoclonal antibody Sipilizumab (α-CD2) to achieve more profound T cell depletion. The B cell depleting agent Rituximab (α-CD20) and prednisone were added after two patients developed donor-specific antibodies. Cyclosporine or Tacrolimus was slowly tapered over several months and completely discontinued at 8 months after confirming freedom from rejection by a 6-month protocol biopsy. All (10/10) patients developed transient chimerism and seven patients achieved tolerance from which four remain completely off immunosuppression (5–13 years) (9, 78, 80). To reduce the toxicity of the regimen and to ameliorate the engraftment syndrome cyclophosphamide has recently been substituted by TBI (2 × 150 cGy). So far two patients have been treated with this modified protocol, from which one remains off immunosuppression for more than 3 years. Future trials are expected to include the use of belatacept, based on murine (81, 82) and non-human primate studies (79, 80, 83).

### Stanford Group

The approach from Stanford University is based on the observation that TLI was sufficient to prolong MHC-mismatched skin graft survival in mice and even to achieve long-term acceptance (>120 days) in combination with bone marrow cells (84). After successful establishment of this approach in larger animals (85, 86), the group went on to the clinical setting resulting in the first well-documented report of actively acquired immune tolerance in humans. Two recipients received 20 fractionated doses of TLI (100 cGy) and six doses of ATG (2 mg/kg; days 0, 2, 4, 6, 8, and 10), however, without hematopoietic stem cell transplantation (87).

Due to limited success rates and their experience from pre-clinical models, the group decided to add hematopoietic stem cell transplantation in 6 HLA-mismatched patients. After kidney transplantation (day 0), the patients received 10 doses of TLI (80–100 cGy; days 1–10) and 6 doses of ATG (1.5 mg/kg; days 0, 1, 3, 5, 9, and 14). CD34^+^ stem cells (3.1–11.1 × 10^6^) were column enriched from G-CSF mobilized blood stem cells, cryopreserved and administered 11 days after kidney transplantation. The cell product contained relatively low numbers of CD3^+^ T cells (<0.1 × 10^6^) to reduce the risk of GvHD. Maintenance therapy consisted of prednisone and cyclosporine. Two patients were weaned off immunosuppression after developing transient chimerism without signs of clinical rejection and exhibiting hyporesponsiveness to donor cells *in vitro*. Both patients developed rejection 3.5 and 5.5 months after withdrawal of immunosuppression. Increasing the dose of TLI did not improve chimerism (11, 88).

Thereupon the Stanford group focused on HLA-matched donor/recipient pairs deploying the same strategy with slight modifications. Patients received 10 doses of 120 cGy TLI and 5 daily doses of ATG (1.5 mg/kg; days 0–4). All patients were infused with column enriched CD34^+^ cells (4.3–17.5 × 10^6^/kg) supplemented with 1 × 10^6^/kg CD3^+^ T cells. Patients featuring stable chimerism for at least 6 months without signs of GVHD or clinical rejection were weaned off immunosuppression. 17 out of 22 patients were successfully tapered off immunosuppression. Seven patients exhibited stable mixed chimerism while the remaining 10 patients lost donor chimerism during or after withdrawal of cyclosporine. One patient with lupus had to return to maintenance immunosuppressive therapy after a systemic lupus flare.

Based on this success, the group recently reattempted their approach with haploidentical donor/recipient pairs. Currently, a dose escalation study is under way in order to determine the optimal dose of CD34^+^ cells and CD3^+^ T cells that would promote persistent mixed chimerism. Ten patients have been enrolled so far revealing that high levels of chimerism (at least 65% at 60 days) can be achieved with 50 × 10^6^/kg CD3^+^ cells and 10 × 10^6^/kg CD34^+^ cells. The ability of these patients to undergo successful drug withdrawal will be subject of a subsequent report with larger numbers of patients (11, 89).

### Northwestern Group

The group from Northwestern University adopted a regimen for patients with non-malignant hematologic diseases and haploidentical donors (90). Kidney recipients received fludarabine (30 mg/m^2^, days −4 to −2) together with dialysis, cyclophosphamide (50 mg/kg, days −3 and 3), and TBI (200 cGy, day −1). One day post kidney transplantation (day 0), a specially designed cell product (FCRx) engineered from G-CSF mobilized blood mononuclear cells is infused. Maintenance therapy in the form of Tacrolimus and mycophenolate mofetil is provided until a control biopsy is performed at 6 months. If the biopsy is clear, renal function is stable and more than 50% donor chimerism is present, immunosuppressive therapy is slowly tapered off. 30 of 31 patients exhibited donor chimerism at 1 month and 19 fulfilled the criteria for discontinuing maintenance therapy. The majority of the tolerant patients exhibited full chimerism (>98%) and three subjects mixed chimerism (10, 62, 73).

**Figure 1:**
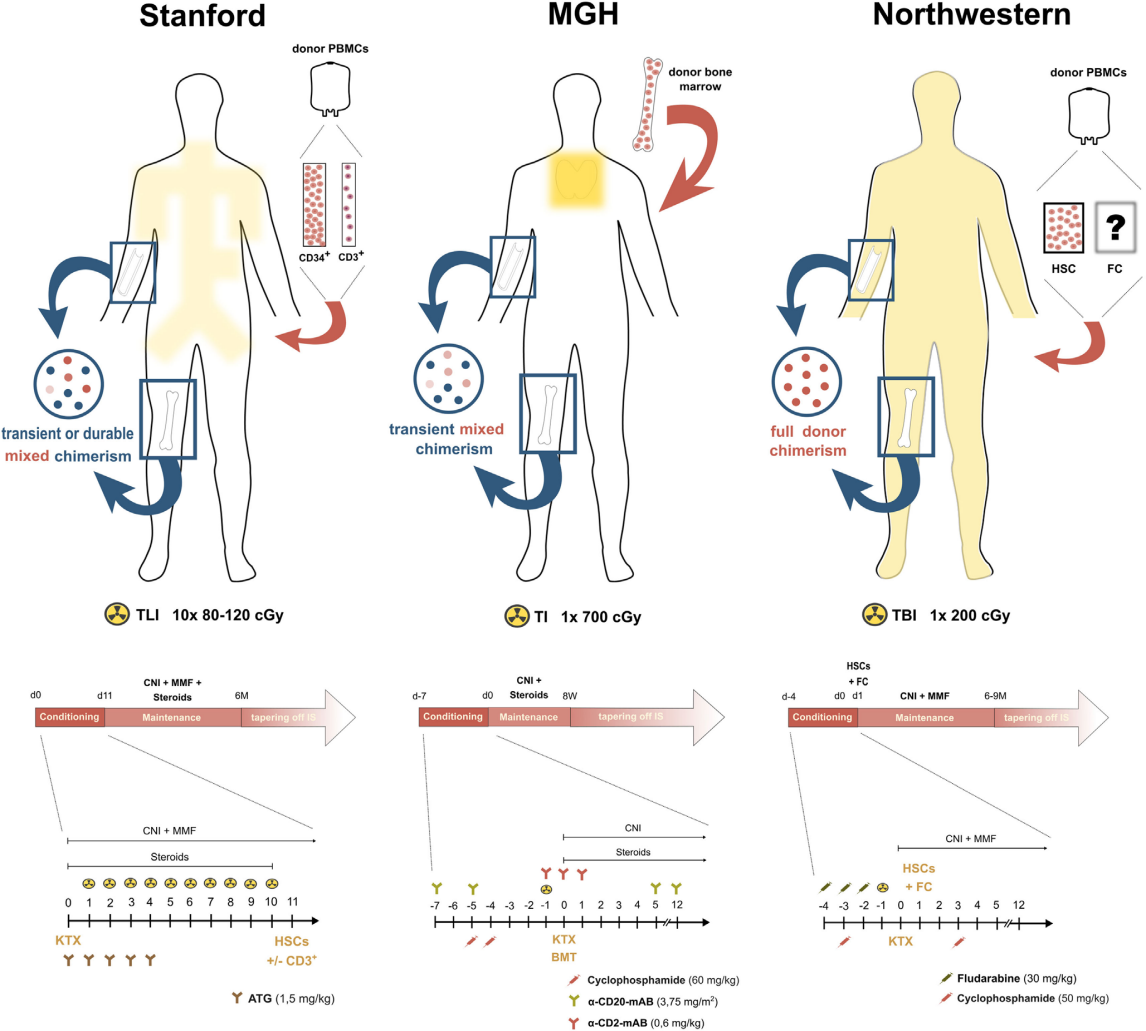
Conditioning strategies in the clinical pilot trials of chimerism-based kidney transplant tolerance. Yellow areas within the silhouettes indicate irradiation fields, the blue circles on the left side display the form of chimerism achieved with each conditioning regimen and the illustration on the right side outlines the administered stem cell product. Below each illustration, an overview about the immunosuppressive course of the respective center is depicted.

**Figure 2:**
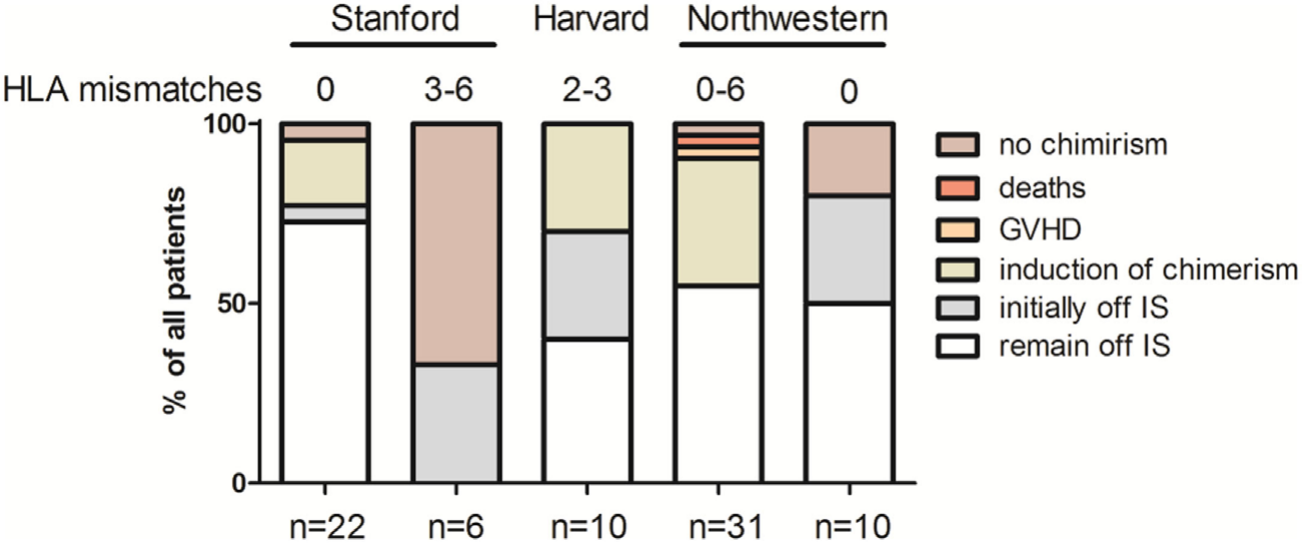
Outcome overview of clinical pilot trials of chimerism-based tolerance in kidney transplantation—graphic illustrations summarizing the key outcomes of the first pilot trials combining hematopoietic stem cell transplantation and kidney transplantation for the purpose of mixed chimerism and tolerance.

The investigators recently initiated a trial in which transplant recipients received multiple infusions of cryopreserved iliac crest and/or CD34^+^ mobilized cells from HLA identical donors without myelosuppressive recipient conditioning, an approach reminiscent of previous bone marrow augmentation trials (91). Iliac crest bone marrow (0.3–1.0 × 10^6^) was infused 5 days post transplantation and GCSF-mobilized CD34^+^ cells were infused 3, 6, and 9 months post transplantation. The recipients received two doses of Alemtuzumab (0.3 mg/kg; days 0 and 4) and maintenance therapy consisted of Tacrolimus and mycophenolate mofetil. No myelosuppressive conditioning was given and, consequently, no macrochimerism was induced, but rather only microchimerism was observed. After 3 months, Tacrolimus was replaced by Sirolimus (Rapamycin) and mycophenolate mofetil was discontinued between 12 and 18 months and Sirolimus after 24 months. Recipients were considered tolerant if they had normal biopsies and renal function after an additional 12 months without immunosuppression. Five of ten patients were successfully withdrawn from immunosuppression and showed normal protocol biopsies at 36 months. Tolerant patients exhibited transient chimerism for the first year and both tolerant and non-tolerant recipients exhibited increased proportions of CD4^+^CD25^high^CD127^−^FOXP3^+^ regulatory T cells and CD19^+^IgD/M^+^CD27^−^ B cells (73, 92) (Figures 1 and 2; Table 1).

**Table 1:** Key parameters of pilot trials combining hematopoietic stem cell transplantation and kidney transplantation for the purpose of mixed chimerism and tolerance.

Human leukocyte antigen-matching	Boston		Northwestern		Stanford		
	Haploidentical	Haploidentical	Mismatched	Matched	Mismatched	Haploidentical	Matched
Number of mismatches	2–3	2–3	0–6	0	3–6	1–3	0
Patients included	*n* = 10	*n* = 2	*n* = 31	*n* = 10	*n* = 6	*n* = 6	*n* = 22
Induction of chimerism	10/10	2	30/31	8/10	2/6	5/10	21/22
Transient mixed chimerism	10	Ongoing	5	8/10	2	3	9
Stable mixed chimerism	0	Ongoing	3	0	0	2	7
Full donor chimerism	0	Ongoing	16	0	0	0	0
Initially off immunosuppression	8/10	1^a^/2	19/31	8/10	2/6	2/6	17/22
Rejection^b^	3	0	0	3	2	0	0
Graft Loss	3	0	2	0	0	0	0
Remaining off IS	4/10	1^a^/2	19/31	5/10	0	0^a^	16^a^/22
Graft-vs.-host disease	0	0	2	0	0	0	0
Death	0	0	1	0	0	0	0

*^a^Ongoing trial*.

*^b^Only patients where IS was withrawn are included*.

## Understanding the Mechanisms of Tolerance

These first pilot trials provide the conceptual framework for the further development of chimerism-based approaches as clinically viable treatment option. In order to refine tolerance inducing strategies, it will be important to understand the underlying mechanisms establishing and maintaining tolerance in greater detail. While the circumstances seem fairly evident in the event of full chimerism, the tolerization of pre-existing recipient T cells in mixed and transient chimeras with an intact T cell repertoire still remains incompletely understood. The group from Stanford conducted mechanistic studies in the murine setting. They found that repeated doses of TLI cause severe DNA damage which drives large numbers of lymphocytes into apoptosis. CD8^+^ dendritic cells are less sensitive to irradiation and possess specialized receptors (Tim-4, DEC205) that recognize products of dying cells. Activated CD8^+^dendritic cells activate the immunomodulatory enzyme indoleamine 2,3-dioxygenase and trigger host NKT cells to produce IL-4. Under these conditions, the interaction of IL-4-producing NKT cells with myeloid-derived suppressor cells and Tregs contributes to the induction of tolerance. Myeloid-derived suppressor cells thwart alloreactive T cell responses through the release of Arginase-1 which deprives local T cells from the essential amino acid l-Arginine and increases the production of superoxide (93). Furthermore, myeloid-derived suppressor cells exhibit an increased surface expression of PDL1 and prompt the residual T cell pool including Tregs to express PD1 (94, 95). The interaction of PDL1 with PD1 attenuates effector T cell functions while promoting the induction, maintenance, and function of Tregs (96). Expression of PD1 on Tregs was also linked to enhanced secretion of IL-10 which preserved the allograft and chimerism (97).

The group from Boston started to investigate the mechanisms sustaining tolerance in patients that had lost chimerism. They found that both circulating and intragraft Foxp3^+^ Tregs were increased in tolerant patients while alloreactive T cells were decreased (98). The increase of peripheral Tregs in the blood resulted from proliferation, thymic emigration, and in one patient from conversion of conventional T cells (99). In order to track alloreactive T cells, they defined a genetic fingerprint of T cells responding to donor cells *in vitro* before transplantation using high-throughput sequencing. Those clones were specifically reduced in tolerant patients but remained unchanged in stable kidney recipients on immunosuppressive therapy (100). Since these patients exhibit transient chimerism, it is likely that donor-reactive T cells were deleted by peripheral mechanisms.

Recently, the group exploited a cohort of tolerant non-human primates that had accumulated over the years to investigate the underlying tolerance mechanisms emerging through transient chimerism in more detail. Tolerant recipients lost anti-donor CD8 T cell response while a considerable number of CD4 T cells proliferated against donor cells *in vitro*. A substantial fraction of these responding CD4 T cells constituted Foxp3 Tregs which were induced from conventional T cells in a TGF-β-dependent manner. The suppressive function of these induced Tregs was contact dependent and inhibiting their induction through TGF-β blockade restored CD8 T cell proliferation *in vitro* (101).

## Future Prospects

As different as the individual approaches may be, they all agree on the pivotal role of Tregs, suggesting that steering the differentiation of naïve CD4 T cells toward regulation is a critical step for the induction of tolerance. The differentiation of naïve CD4 T cells into various T helper subsets is guided by the surrounding cytokine milieu during T cell receptor stimulation. If CD4 T cells commit to a distinct helper lineage, they start to express certain transcription factors that determine their subsequent mode of action such as immunity, tolerance, autoimmunity, or allergy. Therefore, the specific blockade of immunogenic cytokines could be envisioned as suitable approach to drive naïve CD4 T cells into the corner of regulation. This strategy has already been proven successful for the treatment of several inflammatory diseases (102) and it will be interesting to see whether this success will continue in the field of transplantation.

IL-6 is probably the most crucial factor guiding the differentiation of naïve CD4 T cells either toward regulation or inflammation. Inhibition of IL-6 signaling with Tocilizumab is already an established treatment option for autoimmune diseases and currently gains traction in the field of transplantation. Coadministration of Tocilizumab to standard GvHD prophylaxis revealed promising potential in a phase I/II trial (32) and is also tested in several transplant trials (103). In the experimental setting, blocking IL-6 displayed a beneficial effect on the induction of chimerism and tolerance in non-human primates and mice (104, 105).

Alternatively, Treg induction could be promoted by establishing the appropriate cytokine environment. The most vital cytokine for the induction and maintenance of Tregs is IL-2. Nonetheless, current maintenance therapies targeting calcineurin inhibit IL-2 secretion and may, therefore, not be suitable for tolerance-inducing strategies (106). The addition of IL-2 has recently been shown to restore the survival and function of Tregs under calcineurin inhibition and to improve allograft survival (107). Harnessing IL-2 to tip the balance between immunity and regulation is, however, a delicate issue as it is essential for both. The compilation and tissue distribution of the IL-2 receptor probably accounts for its pleiotropic effects. IL-2 preferentially binds to its high-affinity receptor which is constitutively expressed on Tregs and rapidly upregulated on conventional T cells and NK cells upon activation, while resting NK cells and memory CD8 T cells constitutively carry the low-affinity receptor (108). Low doses of recombinant IL-2 have been shown to promote tolerance to islet (109) and skin allografts (110) while high doses of IL-2 abrogated kidney allograft tolerance in non-human primates (111). IL-2/α-IL2 complexes accelerated bone marrow rejection in mice independent of their dose implying that the effect of IL-2 also varies with the transplanted tissue (112).

## Remaining Hurdles

The first clinical pilot trials underpinned the difficulties to achieve durable mixed chimerism over MHC barriers. This issue has often been disregarded in the murine setting where the number of memory T cells is rather low (5–10% of all T cells). Murine models can be improved by enriching recipients with alloantigen primed memory T cells (113) or by using old mice with an increased pool of memory T cells (114). In the human setting, the amount of memory T cells can compromise up to 50% of all circulating T cells (115) from which 1–10% can recognize intact allogeneic MHC molecules through direct allorecognition (116), even in “non-sensitized” recipients through the mechanism of heterologous immunity. Memory T cells are a robust hurdle for tolerance induction due to their lower activation threshold, vigorous effector functions, and their resistance to common immunosuppressive drugs. The presence of memory T cells pre-transplantation has been associated with an increased risk for acute rejection of kidney transplants (117). Pre-exisitng alloreactive memory T cells are most likely generated through recognition of commensal bacteria or environmental antigens (heterologous immunity). Apart from this, memory T cells can occur during homeostatic proliferation following cell depletion or in lymphopenic hosts. Homeostatic proliferation and heterologous immunity have both been shown to impede tolerance induction in mice (118, 119).

Cell-depleting agents enrich memory T cells as they preferentially affect naïve and regulatory T cells (120). T cells with effector/memory phenotype are detectable after Alemtuzumab or ATG induction therapy (120, 121). Moreover, it should be kept in mind that innate immunity which is responsible for the clearance of antibody-coated cells becomes activated (122). Therefore, it would be preferable to block alloreactive cells rather than depleting them. So far CTLA4-Ig (belatacept) is the only costimulation blocker available for clinical use. Besides its availability, CTLA4-Ig also caught attention through its ability to promote chimerism and tolerance in mice (82) and non-human primates (83). Unfortunately, also modern blocking agents have difficulties to keep tab on memory T cells. Terminally effector memory CD4 and CD8 T cells lose CD28 expression and, thus, become resistant to costimulation blockade through CTLA4-Ig (123). Terminal effector T cells are resistant to costimulation blockade even before they lose CD28 expression and their pretransplant frequency has recently been shown to predict episodes of allograft rejection in belatacept-treated patients (124, 125). Increased numbers of CD28^−^ CD4 and CD8 memory T cells have been associated with a poor outcome in renal transplant recipients (126). IL-15 could restore the proliferation of alloreactive CD28^−^ memory CD8 T cells *in vitro* (127). Accordingly, strategies are warranted to block IL-15 signaling under costimulation blockade. Blocking the β-chain (CD122) of the IL-15 receptor has recently been shown to synergize with CTLA4-Ig to prolong allograft survival (128).

Alternative strategies include the use of Alefacept which is a fusion protein consisting of extracellular LFA3 domain and human IgG1. It interacts with CD2 which is upregulated on CD45RO^+^ effector/memory T cells. In this way, Alefacept preferentially targets memory T cells but spares the remaining T cell pool (129). Pre-transplant Alefacept synergized with CTLA4-Ig by targeting CD8^+^ CD28^−^ effector/memory T cells (130). Moreover, blocking the integrins LFA-1 and VLA-4 prolonged skin allograft survival in a mouse model of costimulation blockade-resistant rejection mediated by memory CD8 T cells (113, 131).

## Tolerance or no Tolerance that is the Question

One of the fundamental challenges of tolerance-inducing protocols is to identify those patients in which the protocol was successful and which can, thus, safely be weaned off immunosuppression. Stable multi-lineage chimerism has long been regarded as the most robust predictor for tolerance and donor T cell engraftment in particular as a critical parameter (132). However, the prediction of tolerance becomes more sophisticated in the absence of durable chimerism. Accordingly, there is a high demand for other markers that reliably identify tolerant patients.

The optimal biomarker should allow repeated and non-traumatic measurements that faithfully reflect intragraft processes in real-time with a high precision at affordable prices. The demands are certainly high but advancing biotechnological methods offer unknown opportunities. In the effort to find a reliable biomarker for tolerance, conventional transplant recipients retaining normal kidney function over an extended period of time after withdrawal of all immunosuppressive medication (operationally tolerant) have been compared to healthy subjects or immunosuppressed kidney recipients. Tolerant patients exhibited a specific increase in blood CD4^+^ CD45RA^−^ Foxp3^high^ memory Tregs in comparison to stable kidney recipients and healthy controls. These Tregs exhibited an increased surface expression of GITR and CD39 together with a decreased demethylation of the Treg-specific demethylated region (133). Since only a minor fraction of the whole Treg pool circulates within the blood, the accumulation of Tregs within the graft drew more interest. Indeed, operationally tolerant patients exhibited an increased proportion of Foxp3 Tregs inside their allografts (134). Besides, dissecting the non-regulatory T cell compartment emerged as useful tool to assess alloreactivity. Analyzing the recipients T cell receptor repertoire by high-throughput sequencing becomes increasing popular on that account. Once a genetic fingerprint of donor-reactive T cell clones has been defined, they can easily be tracked over time. The group from Boston could confirm that a reduction of the alloreactive T cell receptor repertoire correlates with tolerance while an increase is associated with rejection (135).

Whole genome microarray-based transcriptional profiling studies additionally revealed B cells as important markers of kidney tolerance (136, 137). These studies led to the disclosure of a distinct B cell signature in operationally tolerant patients and in patients rendered tolerant through hematopoietic stem cell transplantation (138). This specific B cell profile has recently been deployed to identify a cohort of immunosuppressed patients with improved renal allograft function (139). The circumstance that the B cell signature of tolerant patients was conspicuously similar to those of healthy controls raised the question whether these profiles would merely reflect the absence of immunosuppression. Re-evaluation of the data set revealed that common immunosuppressants bias the expression of the investigated genes. Nonetheless, a new gene expression profile could be created that was independent of drug-effects (140). In an alternative approach, tolerant patients could be differentiated from stable patients on the basis of elevated miR-142-3p levels in peripheral blood mononuclear cells. Besides, miR-142-3p expression was stable over time and not affected by immunosuppression (141). The fact that miR-142-3p mainly originated from B cells further substantiated their contribution for tolerance.

In line with this B cell profile, tolerant patients also displayed an increased proportion of naïve (CD20^+^ CD24^low^ CD38^low^) and transitional B cells (CD20^+^ CD24^high^ CD38^high^) in comparison to immunosuppressed kidney recipients (138). In this respect again, it could not be excluded that the redistribution of B cell subsets might result from the absence of immunosuppression. Patients on azathioprine or prednisone namely feature lower numbers of transitional B cells in comparison to patients off drugs (140). On the other hand, the lack of transitional B cells was associated with kidney allograft rejection (142).

No biomarker has emerged yet that has been sufficiently validated to be used for clinical decision making in the routine setting, but progress achieved so far is encouraging and will likely yield success in the future.

## Conclusion

After an extended period of hibernation, the dream of tolerance is gaining increasing attention again. The Holy Grail seems to be in one’s reach but “he who finds the Grail must face the final challenges—three devices of such lethal cunning” (143). In the event of transplantation tolerance, these final challenges would be to reduce host toxicity, to avoid GvHD, and to improve clinical efficacy. Improving tolerance-inducing strategies is a cumbersome and tedious process but the increasing number of patients undergoing such protocols shows that we are heading into the right direction. Besides, novel approaches such as costimulation blockade, Treg therapy, pro-apoptotic molecules, or immunomodulatory antibodies hold out encouraging prospects for the future. Further important steps toward clinical applicability will be to find appropriate biomarkers that reliably predict tolerance.

On the shady side, the burning desire for eternal organ life sometimes makes us forget the involved risks as can be seen by the two recent cases of GvHD. As Goetz von Berlichingen already said in Goethe’s drama: “Where there is bright light, there is also deep shadow.” Throughout the course of history progress has often claimed victims but it remains questionable whether this can be justified for the field of (living donation kidney) transplantation. Be that as it may, the rapidly advancing field of immunology will hopefully allow us to achieve tolerance by safer means. In this regard, the field of tumor immunology emerged as major ally. The mechanisms of how tumors evade host immunity provide vital insights for the design of innovative strategies and could guide future directions (144).

Developing tolerance-inducing strategies has always been and continues to be a highly elaborate process. Therefore, it still remains difficult to assess when tolerance-inducing protocols will reach clinical maturity but there is hope that they will not remain a laboratory solution forever.

## Author Contributions

BM, NG and TW wrote the article and MM revised the manuscript. BM and MM designed the figures and the table.

## Conflict of Interest Statement

The authors declare that the research was conducted in the absence of any commercial or financial relationships that could be construed as a potential conflict of interest.

## Abbreviations

ATG, α-thymocyte globulin; HLA, human leukocyte antigen; MHC, major histocompatibility complex; NK, natural killer; NKT, natural killer T cells; TLI, total lymphoid irradiaton; TBI, total body irradiation; Treg, regulatory T cells.
